# Targeting Dengue Viral Proteins with Eco-Friendly Nanotechnology: A Promising Path for Antiviral Research

**DOI:** 10.3390/v18090995

**Published:** 2026-09-09

**Authors:** D Madan Kumar, R. Raj Bharath, P. Venkataraman, Kumar Sivasubramanian, Leelavathi Prakasam

**Affiliations:** 1 Department of Microbiology, SRM Medical College Hospital and Research Centre, Faculty of Medicine and Health Sciences, SRM Institute of Science and Technology, Kattankulathur, Chengalpattu 603 203, Tamilnadu, India; 2 Department of Transfusion Medicine & Blood Centre, SRM Medical College Hospital and Research Centre, Faculty of Medicine and Health Sciences, SRM Institute of Science and Technology, Kattankulathur, Chengalpattu 603 203, Tamilnadu, India; 3 Division of Medical Research, SRM Medical College Hospital and Research Centre, Faculty of Medicine and Health Sciences, SRM Institute of Science and Technology, Kattankulathur, Chengalpattu 603 203, Tamilnadu, India

**Keywords:** DENV, *Flaviviridae* family, structural proteins, non-structural proteins, green synthesis, nanomedicine, antiviral treatment

## Abstract

Dengue virus (DENV), a mosquito-borne pathogen belonging to the *Flaviviridae* family, continues to be a major global public health concern, causing millions of infections annually across tropical and subtropical regions. The virus encodes both structural and non-structural proteins that play critical roles in its replication, assembly, and immune evasion. Despite substantial research progress, there is still no specific antiviral drug available, and current treatment remains primarily supportive. This review highlights the significance of DENV structural proteins (C, prM/M, and E) and non-structural proteins (NS1–NS5) as potential molecular targets for antiviral interventions. Green-synthesized silver, gold, and iron oxide nanoparticles have demonstrated strong inhibitory activity against DENV by targeting key viral proteins such as the envelope (E), NS3 protease/helicase, and NS5 RNA-dependent RNA polymerase. These nanoparticles interfere with viral entry, replication, and protein synthesis while exhibiting high biocompatibility and minimal cytotoxicity. Collectively, the findings discussed in this review underscore the promise of eco-friendly nanotechnology as a sustainable and effective platform for developing next-generation antiviral therapies against dengue virus.

## 1. Introduction

Dengue virus (DENV) is a mosquito-borne pathogen and a prominent member of the arboviruses—viruses transmitted by arthropods such as mosquitoes and ticks [1,2], which also include Zika virus (ZIKV), chikungunya virus (CHIKV), and West Nile virus (WNV). DENV is a single-stranded, positive-sense RNA virus that belongs to the genus *Orthoflavivirus* within the family *Flaviviridae*. This family includes over 70 additional human pathogens, such as Yellow fever virus and Tick-borne encephalitis virus. *Aedes aegypti* and *Aedes albopictus* mosquitoes serve as the main carriers of dengue fever, a viral infection commonly found in tropical and subtropical regions [3]. DENV is characterized by four antigenically and genetically distinct serotypes: DENV-1, DENV-2, DENV-3, and DENV-4 [4,5], which are responsible for a range of diseases, from dengue fever to more severe manifestations such as dengue hemorrhagic fever (DHF) and dengue shock syndrome (DSS) [6]. Every serotype has the potential to induce the complete range of disease, and contracting one serotype does not confer immunity against the others. The occurrence of secondary infections with a different serotype heightens the risk of severe illness as a result of antibody-dependent enhancement (ADE). Approximately 80% of first dengue virus infections are asymptomatic, but fewer than 20% of infected individuals display clinical manifestations. Dengue fever usually presents with severe headaches, low-grade fever, skin rashes, muscle and joint pain, along with sensations of nausea and vomiting [7]. In 2023, an outbreak of dengue fever occurred in several regions globally, notably including developed nations that had previously seen a low prevalence of the disease [8]. In the past two decades, the incidence of dengue has notably increased, posing a substantial challenge to public health. Between 2000 and 2019, the World Health Organization (WHO) recorded a ten-fold increase in reported cases globally, rising from 500,000 to 5.2 million [9,10]. In 2019, there was an extraordinary surge, with documented cases emerging in 129 countries. Currently, there have been over 5000 reported deaths associated with DENV across more than 100 countries and territories spanning five WHO regions: Africa, the Americas, Southeast Asia, the Western Pacific, and the Eastern Mediterranean [11,12]. Dengue has spread over the world as a result of urbanization, global warming, and increased travel.

Dengue affects all age groups but is particularly severe in children in endemic regions. While live-attenuated vaccines such as Dengvaxia are available, their application is limited due to safety concerns in seronegative individuals. Furthermore, no specific antiviral medication has been discovered so far, meaning current clinical management remains entirely supportive [12,13]. The structural and non-structural proteins of DENV have been studied as targets for antiviral strategies. Nanotechnology offers a transformative platform in antiviral research by enabling targeted delivery, enhanced bioavailability, and controlled release of therapeutic agents. In particular, the emergence of green or eco-friendly nanotechnological methods utilizing plant extracts, biopolymers, and microbial metabolites for nanoparticle synthesis has attracted growing attention due to their biocompatibility, reduced toxicity, and environmental safety. These bioinspired nanomaterials exhibit promising antiviral properties through mechanisms such as viral protein inhibition, disruption of replication processes, and modulation of host immune responses. This research investigates the efficacy of green-synthesized nanoparticles in targeting DENV proteins to inhibit viral replication.

## 2. Investigation of Synthesis Strategies

This article adopts a narrative review methodology to synthesize and critically interpret the current body of knowledge on targeting dengue viral proteins using eco-friendly nanotechnology. Relevant literature was identified through comprehensive searches of established scientific databases, including PubMed, Scopus, and Web of Science. Keywords related to dengue viral proteins (NS1, NS3, NS5, Envelope protein), green or biogenic nanoparticle synthesis, plant-mediated nanotechnology, antiviral nanomaterials, and nanoparticle–virus interactions were employed. Emphasis was placed on peer-reviewed articles that provide mechanistic insights, experimental validation, computational investigations, and translational relevance. Foundational studies and recent advances from the past decade were prioritized to reflect both the evolution and the current state of the field. The review is organized using a thematic framework. First, the biological and therapeutic significance of key dengue viral proteins is discussed, with emphasis on their functional roles in viral replication, host interaction, and immune modulation. Second, the principles underlying eco-friendly nanoparticle synthesis are examined, including plant-mediated, microbial, and biopolymer-assisted approaches. The advantages of green synthesis such as reduced toxicity, enhanced biocompatibility, and environmental sustainability are compared with conventional physicochemical methods. Third, the antiviral mechanisms of green-synthesized nanomaterials are critically evaluated. These include viral entry inhibition, enzymatic suppression, disruption of viral assembly, and immunomodulatory effects. Evidence from in vitro, in vivo, and in silico studies is integrated to highlight structure–activity relationships and protein-specific nanoparticle interactions. The review aims to stimulate interdisciplinary research and guide future efforts toward the development of safe, effective, and sustainable antiviral therapeutics.

## 3. Dengue Viral Genome and Protein Targets

**Figure 1:**
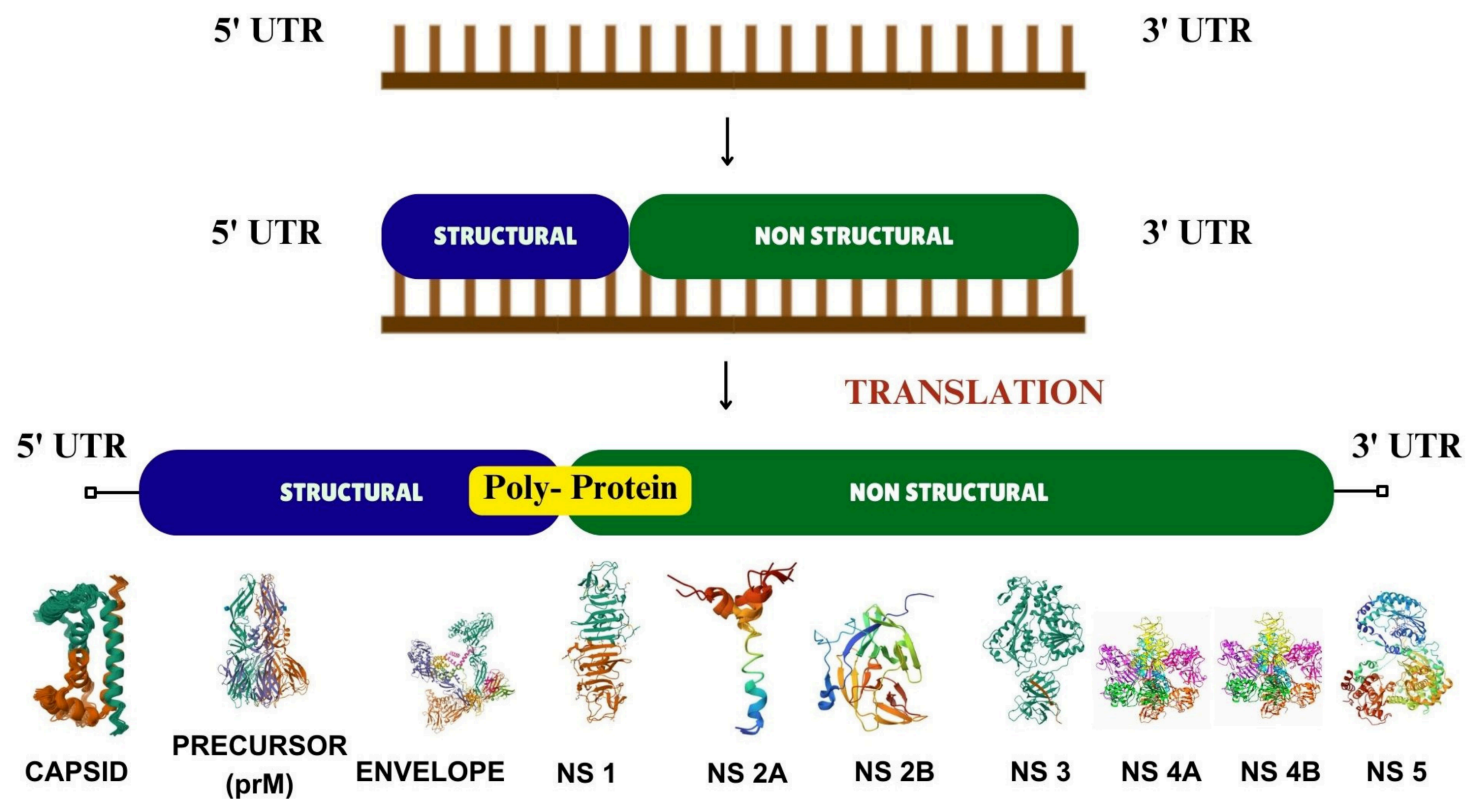
The genome of Dengue virus consists of a single open reading frame (ORF) that encodes a single polyprotein which cleaved into three structural proteins and seven non-structural (NS) proteins.

DENV is characterized by a single-stranded, positive-sense RNA genome of approximately 11 kb [14]. It encodes a singular open reading frame (ORF) that is translated into a polyprotein and subsequently cleaved by host and viral proteases into three structural and seven non-structural proteins (Figure 1) [15,16,17]. The translation and replication of the genome are significantly reliant on its 5′ and 3′ untranslated regions (UTRs) [18,19]. The 5′ UTR features a stem-loop-A (SLA) domain that acts as a facilitator for the RNA-dependent RNA polymerase (RdRp) activity of the non-structural protein NS5 [20,21,22] UTR (~450 nucleotides) contains conserved elements, including a unique dumbbell (DB) structure and a terminal stem-loop (3′ SL), which are essential for viral replication [23]

Structural and Non-Structural (NS) Proteins as Therapeutic Targets:

The three structural proteins Capsid (C), Pre-membrane/Membrane (prM/M), and Envelope (E) are primarily responsible for virion architecture, host cell entry, and maturation:

Capsid proteins: The smallest structural protein (~100 amino acids, 12 kDa) forms an alpha-helical homodimer [24,25]. Its positively charged residues interact with the negatively charged viral RNA to encapsidate the genome, forming the nucleocapsid. Beyond its structural role, the capsid protein has been implicated in modulating host cell processes, such as apoptosis and immune responses, by interacting with specific host proteins including DAXX, core histones (H2A, H2B, H3, and H4), hnRNP-K, nucleolin (NCL), and calcium-modulating cyclophilin-binding ligand [26].

Membrane (M) and Precursor (prM) Protein: The prM protein is co-translationally inserted into the endoplasmic reticulum (ER) membrane and forms a heterodimer with the E protein. It acts as a chaperone, stabilizing the E protein in a prefusion state to prevent premature fusion during intracellular transport [27]. During maturation in the trans-Golgi network, host furin cleaves prM into the mature M protein. Incomplete cleavage can yield partially mature virions that may evade the host immune response and contribute to ADE.

Envelope protein (E): The largest and most immunogenic structural component, the E glycoprotein mediates viral entry and is the primary target for neutralizing antibodies [27,28]. It is organized into three domains: Domain I (DI) acts as a central hinge; Domain II (DII) contains the highly conserved fusion loop for membrane fusion; and Domain III (DIII) is a globular domain responsible for receptor binding [29]. However, the antigenic diversity among the four DENV serotypes, coupled with the risk of ADE, heavily complicates broad-spectrum vaccine design [21,30].

The seven NS proteins collectively form the viral replication complex, manipulate host cell membranes, and suppress host immune responses, presenting multiple targets for antiviral inhibition:

NS1: A multifunctional glycoprotein essential for RNA replication and immune evasion; it interacts with complement pathways, acts as a key biomarker for early diagnosis, and is implicated in vascular leakage [1,31,32,33]

NS2A & NS2B: NS2A coordinates the switch between RNA replication and virion assembly while suppressing interferon α/β responses. NS2B serves as an essential transmembrane cofactor that enhances the protease activity of NS3 [34,35,36].

NS3: A major antiviral target, this large (~70 kDa) multifunctional enzyme exhibits serine protease (in association with NS2B), helicase, and nucleoside triphosphatase (NTPase) activities crucial for polyprotein processing and RNA unwinding [1,34,37,38].

NS4A & NS4B: These highly hydrophobic proteins induce membrane rearrangements to scaffold the replication complex [34,39,40,41]. NS4A promotes viral reproduction by triggering autophagy, protecting infected cells from apoptosis [42], while NS4B inhibits interferon signaling and regulates NS3 helicase activity [43].

NS5: The largest and most conserved flavivirus protein (>100 kDa), NS5 contains a C-terminal RNA-dependent RNA polymerase (RdRp) domain and an N-terminal capping enzyme (MTase/GTase) [1,44,45]. Its critical role in synthesizing viral RNA and degrading host STAT2 makes it a primary focus for drug development.

## 4. Implications for Disease Pathogenesis and Therapeutics

The structural proteins of DENV not only play pivotal roles in the viral lifecycle but also contribute to disease pathogenesis. For instance, the generation of partially mature virions due to incomplete prM cleavage can exacerbate ADE, a condition in which non-neutralizing antibodies increase viral entrance into host cells and cause serious illness consequences such as dengue hemorrhagic fever and dengue shock syndrome [5]. The Envelope protein is responsible for host cell receptor binding and membrane fusion, the Capsid (C) protein is responsible for genome packaging and nucleocapsid formation, and prM/M protein is involved in virus maturation inside host cells. From a therapeutic perspective, targeting the structural proteins offers several opportunities, as shown in Table 1.

## 5. Viral Lifecycle and Vulnerabilities to Nanoparticle Intervention

**Figure 2:**
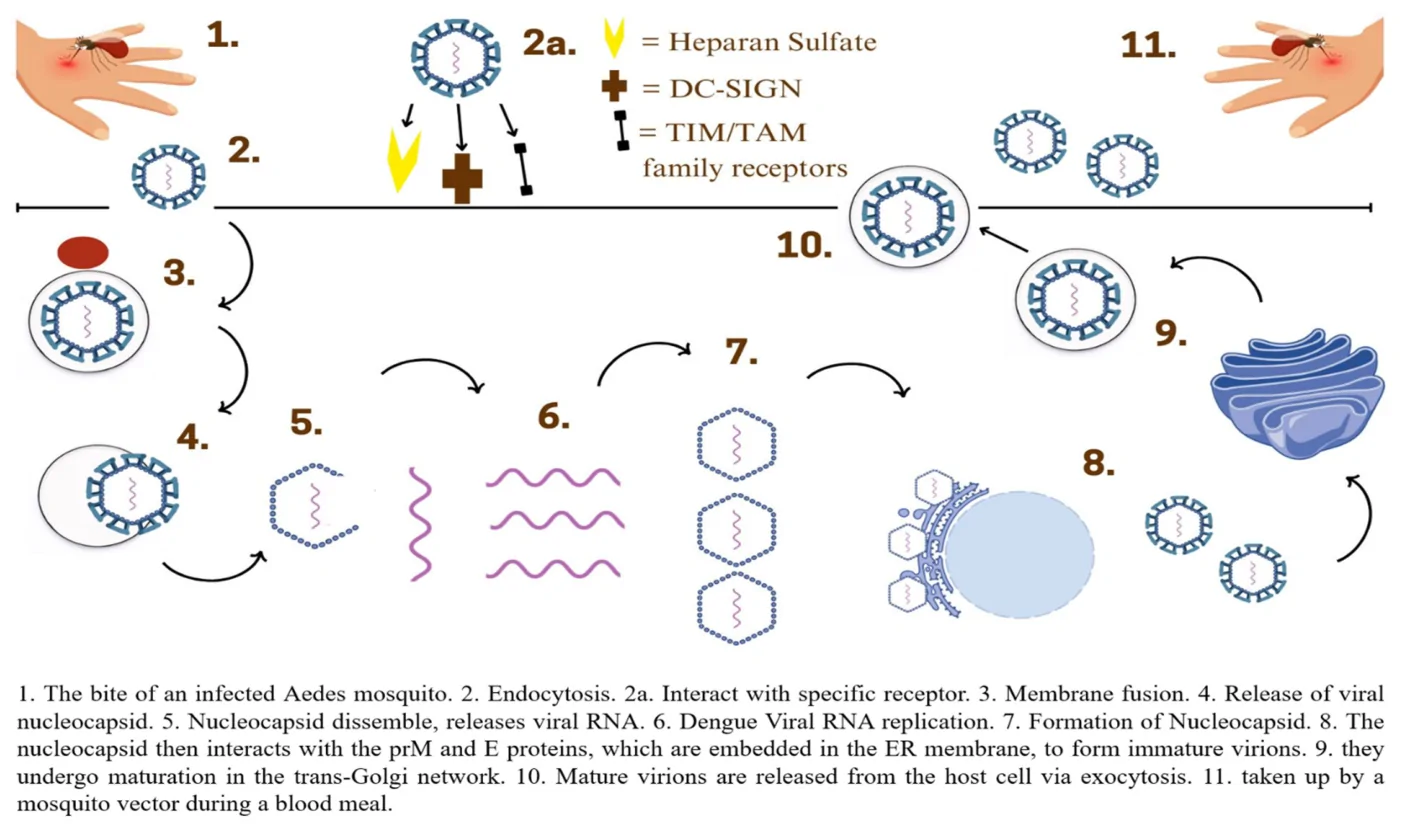
Lifecycle of Dengue Virus.

The DENV life cycle comprises attachment, entry, uncoating, replication, assembly, maturation and release (Figure 2); each presenting unique vulnerabilities for targeted antiviral nanotherapies. The cycle initiates when the viral envelope protein binds to host cell surface receptors, notably Heparan Sulfate, DC-SIGN and TIM/TAM family receptors [48]. Because this E protein-receptor interface is the first point of host contact, it serves as a primary target for green-synthesized nanoparticles designed to sterically block viral docking. Following receptor-mediated endocytosis, the acidic endosomal environment triggers a conformational alteration in the E protein, uncovering its fusion loop in Domain II to facilitate membrane fusion and release the nucleocapsid into the cytoplasm [29,48]. Once uncoated, the single-stranded positive-sense RNA acts directly as mRNA, translating into a polyprotein at the ER [17]. Viral replication then proceeds in ER membrane-bound complexes where the RNA-dependent RNA polymerase (NS5), acting in conjunction with NS3 and NS4A, synthesizes negative-strand RNA as a template for new positive-strand genomes [49,50]. These essential enzymatic processes represent major intracellular targets for nanoparticle-delivered inhibitors. Finally, the newly synthesized viral RNA is encapsidated by the Capsid protein and engages with ER-embedded prM and E proteins to form immature virions [51]. These virions undergo maturation in the trans-Golgi network via prM cleavage; incomplete cleavage can result in partially mature virions that evade immune clearance and contribute to ADE. Mature virions are subsequently released via exocytosis, ready to infect adjacent cells or an *Aedes* vector during a blood meal [1,52].

## 6. Implications for Vaccine and Therapeutic Development

The dual challenges of achieving effective neutralization across all four DENV serotypes and avoiding ADE remain central to vaccine development [53]. Live-attenuated vaccines, such as the Dengvaxia, have shown partial success but are accompanied by safety concerns in seronegative individuals [54]. Structural and non-structural proteins offer promising avenues for next-generation vaccines. Subunit vaccines targeting conserved epitopes on the E protein or incorporating NS1 to elicit robust humoral and cellular responses are under active investigation. In the realm of antiviral therapeutics, inhibitors targeting NS proteins, particularly NS3 and NS5, have shown potential in preclinical studies. High-throughput screening of small molecules and the development of RNA-based therapeutics, such as siRNAs targeting DENV RNA, are promising strategies. Additionally, leveraging host factors co-opted by DENV, such as lipid metabolism pathways, represents an innovative approach to limiting viral replication.

## 7. Nanomedicine

Nanomedicine presents a compelling strategy for addressing arboviruses, which are transmitted by arthropods such as mosquitoes and ticks [55,56]. Arboviruses encompass a range of significant pathogens, including dengue virus (DENV), Zika virus (ZIKV), chikungunya virus (CHIKV), and West Nile virus (WNV). Since there are limited antiviral treatments for these infections, nanomedicine can enhance drug delivery, improve vaccine efficacy, and provide diagnostic tools [57]. Nanoparticles can improve drug efficacy by enhancing bioavailability, protecting drugs from degradation, and targeting infected cells. Certain nanoparticles exhibit intrinsic antiviral properties that can be used to neutralize viruses. Silver nanoparticles (AgNP) have direct antiviral effects by disrupting viral envelopes and inhibiting viral replication. Carbon-based nanomaterials like graphene oxide can block viral entry into cells.

Nanoparticles can be synthesized using various techniques, broadly classified into top-down and bottom-up approaches. The top-down approach involves breaking down bulk materials into nanoparticles using physical or mechanical methods such as ball milling, laser ablation, and sputtering [58]. While effective, these methods often result in polydisperse particles and require significant energy input. In contrast, the bottom-up approach involves assembling nanoparticles from atomic or molecular precursors through chemical and biological synthesis. Chemical methods include sol–gel synthesis, co-precipitation, hydrothermal synthesis, and microemulsion techniques, which provide better control over size and shape. Green synthesis, a biological approach, utilizes plant extracts, bacteria, or fungi to reduce metal salts into nanoparticles, offering an eco-friendly alternative [59].

## 8. Green Synthesis—A Preferred Method for Antiviral Nanoparticles

Green synthesis of nanoparticles, particularly silver nanoparticles (AgNPs), is preferred for antiviral treatment due to several advantages over chemical and physical synthesis methods [60]. Green-synthesized AgNPs are typically less toxic to human cells compared to chemically synthesized ones because they are stabilized by natural biomolecules such as polyphenols, proteins, flavonoids, and alkaloids [61,62,63]. These biomolecules improve the compatibility of AgNPs with biological systems, reducing harmful side effects while maintaining strong antiviral activity. Unlike chemical synthesis, which involves toxic reagents like sodium borohydride or hydrazine. Green synthesis uses natural extracts from plants, fungi, or bacteria. It minimizes environmental pollution and avoids hazardous byproducts. This makes green synthesis an ideal choice for large-scale antiviral applications. Green-synthesized AgNPs exhibit strong antiviral properties due to their unique surface coatings derived from bioactive compounds in plant or microbial extracts [64]. These coatings improve nanoparticle stability and increase their interaction with viral particles, leading to enhanced antiviral effects. Some plant-derived compounds themselves possess antiviral properties, adding synergistic effects. Green synthesis requires fewer resources and is more cost-effective compared to chemical or physical synthesis methods. It eliminates the need for expensive reducing and stabilizing agents, making it suitable for large-scale antiviral treatments [65,66,67]. AgNPs act through multiple pathways. This makes it difficult for viruses to develop resistance, making green-synthesized nanoparticles a long-term solution for viral infections like dengue, influenza, HIV, and COVID-19 [68]. Chemical synthesis often results in toxic residues that can harm human cells, whereas green-synthesized nanoparticles are safer due to their natural stabilizing agents. They exhibit lower cytotoxicity while retaining high antiviral potential, making them suitable for medical applications. Establishing a strong proof-of-concept, recent studies have demonstrated that green-synthesized AgNPs exhibit potent, broad-spectrum antiviral efficacy and excellent host–cell biocompatibility against other significant pathogens, including Foot-and-Mouth Disease Virus (FMDV), Hepatitis A, and Coxsackieviruses [69,70].

Furthermore, the scope of eco-friendly nanotechnology extends beyond direct antiviral mechanisms to encompass integrated disease management. As summarized in Table 2, recent research highlights the multifaceted applications of green-synthesized nanomaterials and raw plant extracts. Because dengue is an arthropod-borne disease, interrupting the transmission cycle is just as critical as antiviral therapy. Consequently, many biogenic nanoparticles exhibit potent dual-action properties—acting not only as virucidal agents against DENV but also demonstrating high larvicidal, pupicidal, and mosquitocidal efficacy against *Aedes aegypti* vectors. Additionally, their broad-spectrum antibacterial properties help prevent secondary opportunistic infections. Table 2 outlines these diverse, eco-friendly investigations, advocating for the holistic use of biological products in global arbovirus control.

## 9. Anti-Dengue Potential of Green Synthesized Silver Nanoparticles

**Figure 3:**
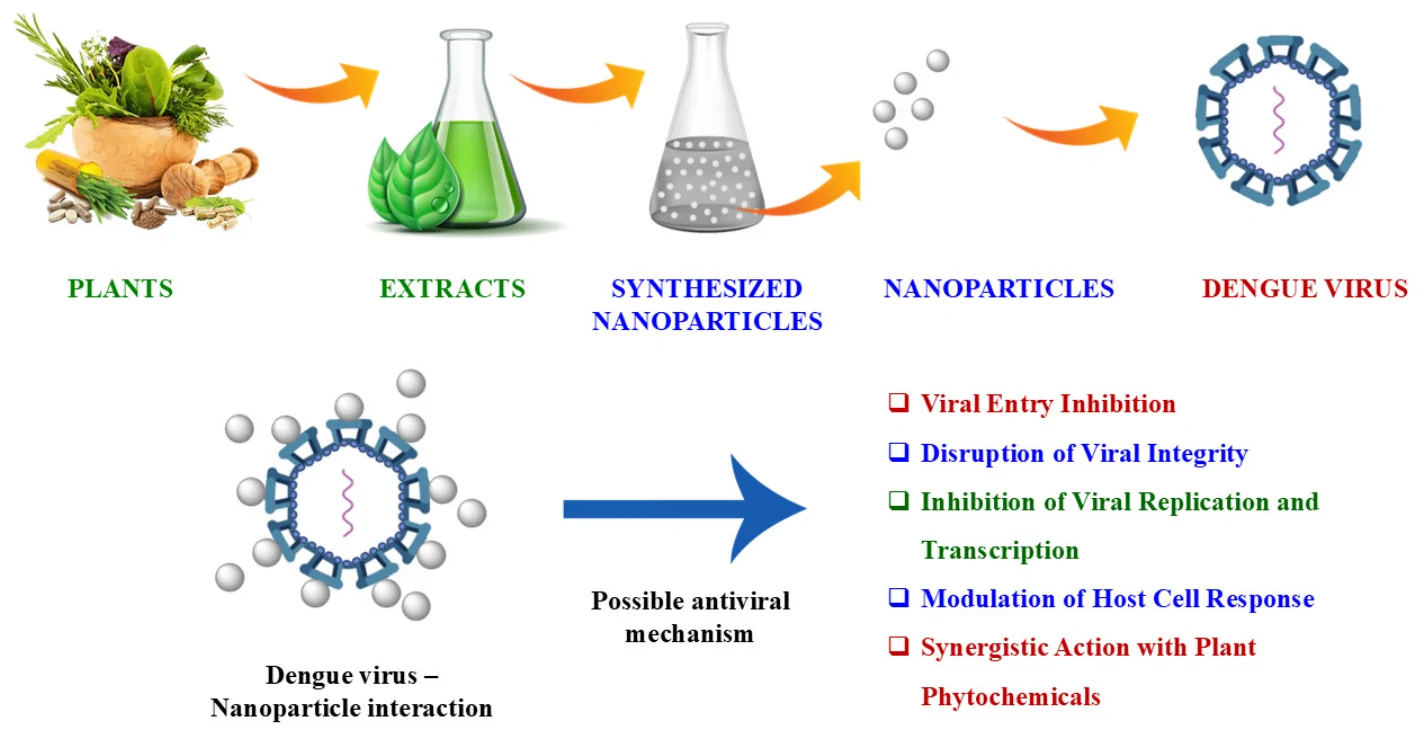
Anti-Dengue potential of green synthesized nanoparticles.

Building upon their broad-spectrum efficacy, biogenic silver nanoparticles (AgNPs) exhibit potent, targeted antiviral effects against the dengue virus. Rather than merely acting as passive agents, these nanoparticles interact directly with DENV particles to disrupt key stages of viral infection, particularly attachment and entry into host cells (Figure 3). The antiviral mechanism against DENV is predominantly size-dependent, with smaller nanoparticles demonstrating increased efficacy by binding viral surface proteins and impeding viral-host cell interactions, thus preventing viral replication. Because they target multiple viral components simultaneously—such as the envelope proteins and viral genetic material—these nanoparticles offer a reduced likelihood of inducing viral resistance. Apart from direct virucidal effects, these nanoparticles also induce localized oxidative stress via reactive oxygen species (ROS) and facilitate host immune modulation.

Several research investigations have reported the anti-dengue properties of biogenic silver nanoparticles against DENV-2. A research study focuses on the biosynthesis of silver nanoparticles (AgNP), which were synthesized using an inexpensive extract from the alga *Centroceras clavulatum*. Assessed their impact on the dengue virus and examined their toxicity in the context of *Aedes aegypti*, the vector responsible for transmitting the dengue virus. The results of the study show that *C. clavulatum*-synthesized AgNP inhibited dengue (serotype dengue virus type-2 (DEN-2)) viral replication in Vero cells. Interestingly, 50 μg/mL of green-synthesized AgNP exhibited no cytotoxic effects on Vero cells, while it significantly reduced DEN-2 viral growth by over 80%; a concentration of 12.5 μg/mL was able to inhibit viral growth by more than 50% [84].

The study [82] demonstrated the biosynthesis of AgNPs using aqueous seed extract of Moringa oleifera as both a reducing and stabilizing agent. The method produced stable, spherical nanoparticles with a mean size of ~100 nm, as confirmed by UV-Vis, SEM, FTIR, EDX, and XRD analyses. The presence of bioactive phytochemicals such as flavonoids, polyphenols, and amides played a key role in nanoparticle stabilization and bioactivity. Silver nanoparticles synthesized via M. oleifera showed minimal cytotoxicity on Vero cells at concentrations up to 40 µg/mL over 48 h. The lack of significant morphological changes or viability loss indicates the biocompatibility of the green-synthesized AgNPs, making them suitable for antiviral applications. Green-synthesized AgNPs exhibited remarkable antiviral activity against dengue virus serotype 2, Viral titer reduced from 7 log10 TCID50/mL to 3.2 log10 TCID50/mL after treatment with 20 µL/mL AgNPs. Plaque assay showed a dramatic reduction in viral yield from 5.8 log10 PFU/mL to 1.4 log10 PFU/mL at 6 h post-infection. Treated cells displayed reduced cytopathic effects and viral load, confirming the inhibitory action of AgNPs on viral replication. These results position green synthesized silver nanoparticles as potential antiviral agents that target early stages of DENV replication. Interestingly, the AgNPs also showed potent larvicidal and pupicidal activity against *Aedes aegypti* with LC_50_ values ranging from 10.24 ppm (1st instar) to 21.17 ppm (pupae). This dual bioactivity against both virus and vector enhance the strategic potential of AgNPs for integrated dengue control programs. Table 3 outlines the evidence related to the targeting of Dengue viral proteins.

## 10. Nanotechnology Based Targeting of the E Protein

The E protein’s accessibility and conservation across DENV serotypes make it a promising universal antiviral target. Eco-friendly nanoparticles can inhibit viral attachment, fusion, and replication processes by interfering with E-protein functionality, gene expression, or conformational dynamics, offering a safe, scalable, and sustainable solution in antiviral nanomedicine development [88]. Gold (AuNPs) and silver nanoparticles (AgNPs) functionalized with sulfonated or polyphenolic ligands can bind electrostatically to the E protein, blocking receptor interaction and viral fusion [89]. Surface plasmon resonance (SPR)-based biosensors using graphene oxide or Fe-nanocellulose composites allow ultrasensitive detection of E-proteins down to picomolar levels, indicating strong sensing interactions between nanomaterials and the viral E surface [90].

Green-synthesized silver nanoparticles derived from *Bruguiera cylindrica*, *Moringa oleifera*, and *Sonneratia alba* extracts have been shown to reduce DENV E-protein expression through transcriptional suppression mechanisms, demonstrating significant viral inhibition in vitro. A research study [83] concluded that silver nanoparticles could be synthesized using an aqueous extract of *Bruguiera cylindrica*, a mangrove species with traditional medicinal value used for anti-dengue applications. The AgNPs were spherical with a size range of 30–70 nm. UV-Visible spectroscopy confirmed synthesis with an SPR peak at 430 nm. FTIR revealed functional groups (amines, carboxylic acids) indicating phytochemical involvement. XRD and SEM-EDX showed crystalline structure and confirmed silver content. At a concentration of 30 µg/mL, AgNPs reduced Vero cell viability by ~35%, indicating moderate cytotoxicity at higher doses. The cytotoxicity profile supports potential antiviral applications within safe concentration ranges. Notably, the treatment with silver nanoparticles demonstrated a reduction in the expression of the dengue viral E-gene, which is responsible for coding the structural envelope (E) protein. The Western blot and RT-PCR confirmed these results. The viral E-gene exhibited a down-regulation in a dose-dependent manner, resulting in a notable decrease in envelope proteins when compared to the control group. These results indicate that AgNPs interfere with viral RNA replication and protein synthesis, potentially suppressing viral replication cycles. Remarkably, the AgNPs also displayed strong larvicidal and pupicidal activity against *Aedes aegypti*, with LC_50_ values ranging from 8.93 ppm (I instar larvae) to 30.69 ppm (pupae). This dual functionality targeting both virus and vector positions B. cylindrica-derived AgNPs as a unique candidate for integrated dengue management. Importantly, exposure to low doses of AgNPs did not negatively impact goldfish (Carassius auratus) predation on mosquito larvae, indicating eco-friendly potential with minimal harm to natural mosquito predators. This study provides robust evidence that green-synthesized AgNPs using Bruguiera cylindrica exhibit potent antiviral activity against dengue virus, coupled with effective vector control and acceptable cytocompatibility. The downregulation of the viral E gene and reduced envelope protein expression underscore their potential as next-generation antiviral agents [83].

Sonneratia alba Sm. derived silver NPs tested in the concentration range of 5 µg/mL to 15 µg·mL^−1^ demonstrated a significant reduction in the Viral E-protein, suggesting a potential anti-dengue effect [91,92]. Comparable outcomes were seen for the silver nanoparticles generated from Sonneratia alba Sm., tested within a concentration range of 5 µg/mL to 15 µg/mL, which demonstrated a significant reduction in the Viral E-protein, suggesting a potential anti-dengue impact [82,91]. Bioinformatics plays a crucial role in identifying excellent drug targets. Recently, significant efforts have been made in the search for effective molecules aimed at targeting various structural or non-structural proteins of the dengue virus [93,94]. The investigation from the study [95] demonstrates that oleanolic acid exhibits the highest binding affinity with both the dengue virus proteins NS1 and NS5, with binding energies of −9.42 Kcal/mol and −8.32 Kcal/mol, respectively. The current investigation demonstrated that the extract of L. cephalotes and its compounds exhibited virucidal activity against the dengue virus, specifically the DENV-2 strain. The docking scoring function of oleanolic acid was measured at 125.11 nano-molar for the NS1 protein, whereas for the NS5 protein, it was recorded at 798.18 nano-molar. The inhibition and electrostatic values were determined to be 798 μM and −0.21 Kcal/mol [95] antivirals offer a sustainable and safer alternative to synthetic drugs in the management of dengue fever. Plant-based and natural antivirals show promising antiviral activity with minimal side effects and lower toxicity compared to conventional chemical drugs [74].

There are several potential mechanisms by which green synthesized nanoparticles may function as antiviral agents, including the ability of silver nanoparticles to disrupt the initial stages of DENV infection. The AgNPs interact with the viral envelope (E) protein, which mediates attachment and fusion with the host cell membrane. This binding causes conformational changes or steric hindrance, blocking the virus from docking to cellular receptors. These can also bind to receptors on the host cell (e.g., heparan sulfate proteoglycans, DC-SIGN), preventing virus-receptor interaction. AgNPs may directly destabilize the virion structure through physical and chemical interactions through generation of Reactive Oxygen Species (ROS) which damages viral membranes and nucleic acids and Membrane Disruption, where AgNPs can penetrate or destabilize the lipid envelope of the virus, leading to loss of infectivity. Phytochemicals used in green synthesis (e.g., catechins, quercetin, gallic acid) exhibit independent antiviral properties, including binding to viral RNA or proteins and Enzyme inhibition (e.g., NS2B/NS3 protease inhibition). When combined with silver nanoparticles, Synergistic effects lead to higher antiviral potency, reduced required dosage and reduced toxicity to host cells due to biocompatible capping layers.

## 11. Antiviral Target of NS3 & NS5 Protein

Structurally, the NS3 helicase domain contains conserved motifs for nucleotide binding and hydrolysis, classified under superfamily 2 of RNA helicases. It unwinds double-stranded RNA during replication by coupling NTP hydrolysis to mechanical RNA strand separation, a critical step in the viral life cycle. Mutations or inhibitors disrupting NS3′s helicase or protease functions impair viral replication, making it an attractive antiviral target. Inhibitor design for NS3 focuses on blocking its protease active site or interfering with helicase activity. Given its multifunctionality and conservation among flaviviruses, NS3 is a promising target for broad-spectrum antivirals. Eco-friendly nanotechnology approaches can exploit this target by using green-synthesized nanoparticles to deliver NS3 inhibitors or by designing nanomaterials that directly interact with and inhibit NS3 enzymatic activities, disrupting viral replication [96].

NS5 also participates in immune evasion by interacting with host proteins to downregulate interferon responses. Structurally, the MTase and RdRp domains are connected by a short linker influencing overall protein conformation. The conserved architecture among flaviviruses allows for designing drugs effective against multiple viruses. Targeting NS5 with antiviral agents aims at inhibiting either the methyltransferase or polymerase functions, effectively halting viral genome replication. Nanotechnology can enhance this targeting by using biogenic nanoparticles for specific delivery of NS5 inhibitors or direct interaction with NS5 to block its enzymatic functions, thereby impeding viral replication and assembly. NS5 also forms complexes with NS3 during replication, highlighting the potential for combination targeting strategies. Eco-friendly nanomaterials synthesized from plant extracts and other green methods provide a sustainable means of developing such antiviral interventions with reduced environmental impact [97,98].

## 12. Challenges and Limitations in Clinical Translation

While green-synthesized nanoparticles show significant anti-dengue potential, several critical hurdles must be addressed before clinical translation.

Paucity of In Vivo Data: Currently, the literature relies heavily on in vitro cell line models, which show excellent phenotypic viral inhibition and biocompatibility. However, translating these findings to in vivo success remains a significant challenge. The limited in vivo studies conducted thus far in related nanomedicine fields suggest that while green-synthesized nanoparticles can successfully circulate and reduce viral loads in animal models, their pharmacokinetic behavior—including biodistribution, bioavailability, and systemic clearance—often diverges from in vitro predictions due to complex interactions with host serum proteins (protein corona formation). Rigorous in vivo mammalian trials are urgently needed to correlate in vitro dosage efficacy with actual physiological outcomes and to establish standardized therapeutic windows.

Lack of Mechanistic Depth: Although phenotypic viral inhibition is well-documented, precise molecular mechanisms often remain poorly characterized. Advanced analytical studies are required to map the exact binding kinetics and intracellular fate of these nanoparticles, rather than merely inferring downstream effects.

Standardization of Green Synthesis: The inherent variability in phytochemical composition—where the concentration of active flavonoids or capping agents naturally fluctuates based on harvest season and extraction protocols—complicates the reproducible synthesis of nanoparticles with consistent sizes, shapes, and surface charges.

Long-Term Toxicity and Bioaccumulation: Despite demonstrating low in vitro cytotoxicity, the risks of chronic exposure and long-term bioaccumulation of metal nanoparticles in mammalian organs remain largely unexplored. Comprehensive safety profiles mapping metabolic degradation pathways are essential.

## 13. Conclusions

The integration of eco-friendly nanotechnology into antiviral research represents a transformative approach to combating dengue virus infections. Green-synthesized nanoparticles, derived from plant and microbial sources, offer an alternative to conventional chemical synthesis methods. Their unique physicochemical properties enable precise targeting of dengue viral proteins, particularly the non-structural and envelope proteins, thereby disrupting key viral replication and assembly mechanisms. Current in silico and in vitro studies demonstrate the strong antiviral potential of various green-synthesized metal and metal oxide nanoparticles, highlighting their ability to interact with critical viral targets while minimizing cytotoxic effects on host cells. However, despite these promising findings, significant challenges remain in translating laboratory results into clinical applications. Standardization of synthesis protocols, comprehensive toxicity profiling, and mechanistic studies at the molecular level are essential to ensure safety and efficacy.

In summary, eco-friendly nanotechnology stands as a promising frontier in the design of novel antiviral strategies against dengue virus. This comprehensive review suggests that among all the proteins, Envelope protein, NS3 and NS5 nonstructural proteins are considered the most effective targets for antiviral nanotechnology because they are highly conserved across all dengue virus serotypes and essential for viral replication. Green-synthesized nanoparticles and phytochemical conjugates have demonstrated strong inhibitory potential against these proteins, offering durable resistance and cross-serotype efficacy. Continued interdisciplinary research combining nanotechnology, virology, and computational biology will be crucial to harness its full therapeutic potential and pave the way for next-generation antiviral interventions that are both effective and environmentally sustainable.

## Figures and Tables

**Table 1:** Summary of therapeutic strategies and diagnostic applications for viral Envelope and Membrane proteins.

Target	Therapeutic Approach	
Envelope protein	Vaccines	The E protein is a prime candidate for vaccine development due to its immunogenicity and role in viral entry. Efforts such as the development of chimeric vaccines and subunit vaccines focus on eliciting strong neutralizing antibody responses against the E protein [46,47].
	DIAGNOSTICS	Structural proteins, particularly the E protein, serve as biomarkers for diagnostic assays.
Membrane protein and its precursor	Antiviral strategies	Small molecules or antibodies that disrupt the interaction between prM and E, or inhibit the cleavage of prM, could prevent the maturation of infectious virions [46].
Capsid protein	Antiviral strategies	Small molecule inhibitors (such as ST-148) and peptide mimics targeting the capsid protein can potently disrupt nucleocapsid assembly, prevent viral RNA encapsidation, and block essential interactions with host lipid droplets [26].

**Table 2:** Research investigations highlight the eco-friendly approach in viral treatment.

Research	Nanoparticle/Extracts	Reducing Agent	Application	Targeted Organism	Type of Study
Balasubramanian Swathi 2025 [71]	AgNp	β-Caryophyllene AgNP	larval control	*Aedes aegypti* larvae	Invitro
Khan, M.Z., Oneeb, M., Tariq, K. et al. 2025 [72]	AgNp	*Fusarium oxysporum*, *Metarhizium anisopliae*, and *Trichoderma viride*	larval control	*Aedes aegypti* and *Cx. Pipiens* mosquitoes	Invitro
Wilson, J.J., Mahalakshmi, S., Thangaraj, R. et al. 2025 [73]	AgNp	*Klebsiella pneumoniae*	Antibacterial and Larvicidal	*Anopheles stephensi*, *Aedes aegypti*, and *Culex quinquefasciatus* fourth instar larvae. pathogenic microorganisms such as *Proteus mirabilis*, *Escherichia coli*, *Serratia* sp., *Pseudomonas aeruginosa*, and *Staphylococcus aureus*	Invitro
Selvam, K., Sudhakar, C., Ragu Prasath, A. et al. 2025 [74]	Leaf extract	*Andrographis paniculata* extract	Antiviral and Antibacterial	Dengue Virus, *Staphylococcus aureus* and *Pseudomonas aeruginosa*	Insilico
Rajaganesh, Rajapandian et al. 2024 [75]	iron oxide nanoparticles	*C. aerea* synthesized Mn-doped superparamagnetic iron oxide nanoparticles (CA-Mn-SPIONs)	Anti-dengue potential and mosquitocidal effect	Dengue Virus and *Aedes aegypti* larvae	Invitro and Insilico
Kovendan, Kalimuthu et al. 2024 [76]	AgNp	*Malvastrum coromandelianum*	Anti-dengue potential and mosquitocidal effect	Dengue Virus and *Aedes aegypti* larvae	Invitro and Insilico
Sulochana Kaushik et al. 2020 [77]	Leaf extract	*Cyamopsis tetragonoloba* (Gaur) L. supercritical extract	Anti-dengue activity	Dengue Virus	Invitro and Insilico
Antonia W Bere et al. 2021 [78]	AgNp	*Carica papaya* Leaf Extract	Anti-dengue potential	Dengue Virus	Invitro
Sathiyapriya Renganathan et al. 2018 [79]	AgNp	*Carica papaya* Leaf Extract	Anti-dengue activity	Dengue Virus	Insilico
Vikrant Sharma et al. 2019 [80]	AgNp	*Andrographis paniculata*, *Phyllanthus niruri*, and *Tinospora cordifolia*	Antiviral treatment	Chikungunya virus	Invitro
Sekaran Muthamil Selvan et al. 2018 [81]	CuO NPs	*T. procumbens*	mosquitocidal effect	*Aedes aegypti* larvae	Invitro
Vasu Sujitha et al. 2015 [82]	AgNp	*Moringa oleifera* seed extract	Anti-dengue activity and mosquitocidal effect	Dengue Virus and *Aedes aegypti* larvae	Invitro
Kadarkarai Murugan et al. 2015 [83]	AgNp	*Bruguiera cylindrica*	Anti-dengue activity and mosquitocidal effect	Dengue Virus and *Aedes aegypti* larvae	Invitro

**Table 3:** Dengue viral proteins and their targeting evidence.

Viral Protein	Function	Nanotechnology Targeting Evidence
NS1	Vital for immune evasion and viral replication. Present both intracellularly and secreted during infection.	Gold and silver nanoparticles have been used to design nanobiosensors and virucidal formulations that bind to NS1, inhibiting replication and improving diagnostic sensitivity [85].
NS3 Protease/Helicase	Controls viral polyprotein cleavage and RNA unwinding, essential for replication.	Green-synthesized nanoparticles carrying phytochemicals can inhibit enzymatic activity of NS3. Protein–protein interaction between NS3 and NS5 is a key antiviral target [86].
NS5 RNA-Dependent RNA Polymerase (RdRp)	Catalyzes viral RNA synthesis and contributes to immune evasion.	Plant-based nanocarriers and biogenic nanoparticles have been shown to inhibit NS5 through computational docking and in vitro assays [86].
Envelope Protein	Mediates viral entry and fusion with host cells.	Eco-friendly lignin-derived and plant-based nanoparticles exhibit potent binding to E protein, blocking viral entry [87].

## Data Availability

No new data were created or analyzed in this study. Data sharing is not applicable to this article.
